# Efficacy and Safety of Low-Molecular-Weight Heparins As An Adjunct to Thrombolysis in Acute ST-Elevation Myocardial Infarction

**DOI:** 10.2174/157340308783565438

**Published:** 2008-02

**Authors:** Andrea Rubboli

**Affiliations:** Cardiac Catheterization Laboratory, Division of Cardiology, Maggiore Hospital, Bologna, Italy

**Keywords:** ST-elevation acute myocardial infarction, enoxaparin, dalteparin, reviparin, unfractionated heparin.

## Abstract

A 48-hour course of intravenous unfractionated heparin (UFH) is the standard of treatment in conjunction with fibrin-specific thrombolysis in ST-elevation myocardial infarction (STEMI). In recent trials, the efficacy and safety of in-hospital administration of subcutaneous low-molecular-weight heparins (LMWH), previously proven effective in non-ST-elevation acute coronary syndromes, have been investigated in the setting of STEMI. The aim of this review was to evaluate the available evidence supporting the use of LMWH in STEMI.

Overall, about 27,000 patients treated with various thrombolytic regimens, were included in 12 open-label randomized clinical trials, where dalteparin, reviparin or enoxaparin were administered. While acknowledging the wide variability in study dimensions, designs and end-points, a higher efficacy of LMWH was observed overall as compared to placebo, and also to UFH (mainly as regards the occurrence of reinfarction). As regards safety, bleedings were more frequent than placebo and comparable to UFH in LMWH groups, with the exception of the pre-hospital ASSENT-3 PLUS trial, where in elderly patients, enoxaparin had an incidence of intracranial hemorrhage twice higher than UFH. In a recent double-blind, randomized, mega-trial including over 20,000 patients, the superior efficacy on in-hospital and 30-day adverse cardiac events (namely reinfarction), and comparable safety on intracranial bleedings, of enoxaparin compared to UFH, was shown.

In conclusion, in-hospital subcutaneous administration of dalteparin, reviparin and enoxaparin, as an adjunct to various thrombolytics in STEMI, appears feasible and at least as effective and safe as 48-hour intravenous treatment with UFH. In accordance with the available strongest evidence, an initial intravenous bolus of enoxaparin followed by twice daily subcutaneous administration for about 1 week should be the preferred regimen, and should be strongly considered instead of intravenous UFH. Along with its easiness of use, not requiring laboratory monitoring, subcutaneous administration of LMWH following STEMI treated with thrombolysis allows extended antithrombotic treatment, while permitting early mobilization (and rehabilitation) of patients.

## INTRODUCTION

Rapid and complete dissolution of the occlusive coronary thrombus is the objective of thrombolytic treatment in acute ST-elevation myocardial infarction (STEMI). Upon clot lysis however, thrombin is exposed and further clot formation is promoted [1]. In addition, plasmin mediated activation of the coagulation system has been shown to be important, as judged by plasma increases of markers of prothrombin activation and thrombin activity (Eisenberg *et al*, JACC 2002). Thrombin induces platelet activation, fibrinogen conversion to fibrin and cross-linking of fibrin chains, therefore causing resistance to clot lysis and propensity to recurrent coronary thrombosis which can be at least partially inhibited by antithrombin agents. However, extensive plasmin activity also induces proteolysis of fibrinogen and coagulation factors which inhibit thrombosis by interfering with fibrin polymerization and platelet aggregation thus exerting an anticoagulant effect. The balance between prothrombotic and anticoagulant effects of thrombolytics is different for fibrin-specific as compared with non fibrin-specific agents being the extent of fibrinogen degradation more pronounced with the latter than with the former [3]. Accordingly, a 24 to 48-hour course of intravenous unfractionated heparin (UFH) is currently recommended (Class I recommendation) as an adjunct to thrombolysis with fibrin-specific agents (alteplase, reteplase, or tenecteplase), whereas such recommendation is less compelling (Class IIb recommendation) when streptokinase is given instead [3, 4].

UFH consists of an heterogeneous mixture of polysaccharide chains with a molecular weight of 3,000 to 30,000 daltons, and acts as an anticoagulant by activating anti-thrombin. This activation resides on a specific pentasaccharide sequence which has high affinity for antithrombin and is randomly dispersed within the heparin molecule. Whereas UFH chains of any length containing this unique sequence can bind to antithrombin and promote inactivation of factor Xa, the formation of the ternary complex of UFH-anti-thrombin-thrombin necessary to enhance inhibition of thrombin (factor IIa) can occur only with UFH molecules with > 18 saccharide units. Since UFH is primarily composed of chains with > 18 units, the anti-Xa:anti-IIa potency is approximately 1:1. On the contrary, low-molecular-weight heparins (LMWH), which are obtained by chemical or enzymatic depolymerization of UFH, are enriched of shorter chains, with a mean molecular weight of 5,000 daltons, and preferentially inhibit factor Xa. Such action, obtained again by the formation of a LMWH-antitrombin complex, is not chain-length dependent, as it is for factor IIa. As a consequence, LMWH act more upstream than UFH in the coagulation cascade, and are therefore more efficient. In addition, LMWH as compared to UFH bind significantly less to plasma proteins, are less neutralized by platelet factor 4, and are associated with less side effects, such as heparin-induced thrombocytopenia and osteoporosis. Finally, LMWH have increased bioavailability and dose-dependant clearance, which make the anticoagulant response more predictable and routine laboratory monitoring unnecessary.

Because of these potential advantages, LMWH have been evaluated in recent years as an adjunct to thrombolysis in STEMI, and are currently recommended as an acceptable alternative to UFH (Class IIb recommendation), provided that patients are less than 75 years of age and significant renal dysfunction (serum creatinine > 2.5 mg/dl in men or 2.0 mg/dl in women) is absent [3, 4].

In this review, the studies of LMWH in combination with fibrinolytics which are at the basis of current recommendations are examined. Also, newer evidence on this issue, which has recently become available, is reviewed.

## CLINICAL TRIALS (TABLE **1**)

The clinical studies of LMWH with thrombolytic treatment in STEMI highly differ in design, compound evaluated, thrombolytic used, doses and duration of treatments, and endpoints. Because of these reasons, as well as of the different pharmacokinetic and pharmacologic properties, the various LMWH are reviewed separately, and a cumulative analysis is subsequently attempted.

### Dalteparin

In the FRAMI study [5], 776 patients with anterior STEMI and receiving streptokinase, were randomized to dalteparin or placebo. The primary endpoint was the composite incidence of left ventricular thrombosis and arterial embolism. Upon evaluation of only the patients who continued treatment (64% of dalteparin and 70% of placebo patients), the occurrence of primary endpoint was significantly reduced by dalteparin, with no significant effect on the reinfarction and mortality rates, but with a significantly higher risk of both major and minor bleedings.

The BIOMACS study [6] evaluated 101 patients treated with streptokinase, who were randomized to dalteparin or placebo. The primary outcome, as represented by TIMI 3 flow grade at 20-28 hours, did not significantly differ between the two groups, although TIMI 0-1 flow grade, and its combination with intraluminal thrombus, were significantly less frequent in the dalteparin group. As regards clinical events, a significantly less incidence at both 24 hours (excluding the initial 6 h when unstable coronary blood flow is common) and 7 days was observed with dalteparin, in the absence of differences in the major/minor bleeding rate.

The ASSENT PLUS trial [7] compared dalteparin with UFH as an adjunct to alteplase in 1639 patients, aiming at evaluating TIMI 3 flowgrade at 4-7 days (primary end-point). Achievement of TIMI 3 flow grade, which was evaluated in 86% of patients, was similar in both groups, whereas reinfarction rate at 7 days was significantly less with dalteparin. Such difference however, was not sustained at 30 days. Finally, no differences in bleedings were reported.

### Reviparin

In the CREATE trial [8], 15,570 patients were randomized to reviparin or placebo as an adjunct to thrombolysis. The two composites primary end-points of death, reinfarction or stroke and previous plus recurrent ischemia with electrocardiogram changes were significantly reduced at 7 days with reviparin. These benefits were persistent at 30 days, when significant reductions in mortality and reinfarction rates were also present. No significant differences were observed in stroke rates. At 7 days, an increase in life-threatening and major bleeding was observed with reviparin, although such a small absolute increment (1 event/1000 treated patients) should be acknowledged, especially when the reduction of both primary end-point (18 fewer events/1000 treated patients) and mortality (15 fewer events/1000 treated patients) are considered.

### Enoxaparin

Glick *et al*. [9], randomized 103 patients to either subcutaneous enoxaparin or placebo for the next 25 days, following treatment with aspirin, streptokinase and UFH for the first 5 days. The primary end-point of combined occurrence of unstable angina, reinfarction and death at 6 months was significantly reduced in the enoxaparin group, mainly as a consequence of the decreased reinfarction rate, since the occurrence of unstable angina and death were comparable.

In the HART-II study [10], 400 patients receiving aspirin and accelerated rt-PA, were randomized to enoxaparin or UFH. The primary end-point was the IRA patency at 90’ and 5-7 days, while the secondary end-point was the occurrence of major bleedings. At 90’ TIMI 2-3 and TIMI 3 flow grades in the infarct-related artery (IRA) did not significantly differ in the two groups. Also, the reocclusion rate at 5-7 days was comparable, although a clear trend favoring enoxaparin was evident. Major bleedings occurred with similar frequency in both groups.

In the ASSENT-3 trial [11], 6095 patients treated with aspirin were randomized to: 1) full-dose tenecteplase and enoxaparin; 2) half-dose tenecteplase with weight-adjusted low-dose UFH and abciximab; 3) full-dose tenecteplase and weight-adjusted UFH. Primary end-points were the composites of 30-day mortality, in-hospital reinfarction/ refractory ischemia (efficacy end-point), and the above end-points plus in-hospital intracranial hemorrhage/major bleedings (efficacy plus safety end-point). In association with full-dose tenecteplase, enoxaparin was significantly more effective on the primary efficacy end-point, as well as on the primary efficacy plus safety end-point. The association of abciximab and UFH influenced both efficacy and efficacy plus safety end-points comparably to enoxaparin and superiorly to UFH. Enoxaparin significantly reduced in-hospital reinfarction and refractory ischemia, along with in-hospital death/reinfarction. Major hemorrhagic complications were not significantly different with enoxaparin as compared to UFH.

The ENTIRE-TIMI 23 study [12] was carried out on 483 patients to determine the effect on the 60’ patency rate of the IRA of 4 pharmacologic regimens, including the three evaluated in the ASSENT-3 trial [11] plus an additional one with half-dose tenecteplase associated with enoxaparin and abciximab. The 4 regimens were similarly effective on the primary end-point of IRA TIMI 3 flow grade at 60’, which was about 50% in all groups. When pooling the results of the different groups according to heparin treatment, the IRA TIMI 3 and TIMI 2-3 flow grade rates were comparable. A favorable trend towards a complete ST-segment resolution at 180’ was observed in enoxaparin groups. Evaluation at 30 days by a blinded Clinical Events Committee of the clinical efficacy end-points showed significantly less death/reinfarction with enoxaparin, when administered with full-dose tenecteplase. This was mainly due to the reduction in reinfarction, which could also be observed when pooling all enoxaparin *vs* all UFH patients. No effect of the two heparin regimens was apparent with the combination treatments including abciximab. Through 30 days, the occurrence of major bleedings was similar in both groups treated with full-dose tenecteplase, regardless of the heparin regimen used. When abiciximab was added, a trend towards a higher bleeding rate was observed with enoxaparin as compared to UFH. Such a trend was also apparent for enoxaparin when pooling patients with respect to the heparin regimen adopted.

Baird *et al*. [13] enrolled 300 patients receiving streptokinase or anistreplase (but not aspirin, which was given only at the end of the investigated treatment), who were randomised to either enoxaparin or UFH. The primary end-point was the occurrence at 90 days of the composite of death, reinfarction or rehospitalization due to unstable angina. Enoxaparin was significantly more effective than UFH, leading to a 30% relative risk reduction of death, reinfarction or recurrent angina. This effect was obtained through a consensual reduction of any single component of the composite end-point. Significant bleeding occurred comparably in the two treatment groups.

In the AMI-SK study [14], 496 patients treated with aspirin and streptokinase were randomized to enoxaparin or placebo. The primary end-point was the IRA patency rate at 5-10 days, while secondary end-points were ST-segment resolution at 90’ and 180’ and occurrence of combined death, reinfarction, recurrent angina and major bleedings at 30 days. Enoxaparin was significantly more effective on the primary end-point and complete ST-segment resolution both at 90’ and 180’. Clinical events at 30 days were also significantly reduced in the enoxaparin group, mainly as a consequence of the reduction of reinfarction. Major hemorrhages were more frequent with enoxaparin, although this difference was not statistically significant.

In the ASSENT-3 PLUS study [15], 1639 patients were randomly assigned in a pre-hospital setting to treatment with tenecteplase and either enoxaparin or weight-adjusted UFH. The primary end points were: composite of 30-day mortality or in-hospital reinfarction/refractory ischemia (efficacy end point) and composite of the previous plus in-hospital intracranial hemorrhage/major bleedings (efficacy plus safety end point). Enoxaparin was comparable to UFH on both the primary efficacy and efficacy plus safety end points. Analysis of the individual components of the end points showed a reduction in in-hospital reinfarction and refractory ischemia rates, but an increase in total stroke and intracranial hemorrhage with enoxaparin. The increase in intracranial hemorrhage however, was seen exclusively in patients over 75 years of age.

The ASENOX study [16], included 633 consecutive patients who received aspirin and were randomly assigned to either: 1) accelerated streptokinase plus enoxaparin (ASKENOX group = 165 patients); 2) accelerated streptokinase plus UFH (ASKUFH group = 264 patients) or 3) regular streptokinase plus UFH (SSKUFH group = 204 patients). When considering the 429 patients in the ASKENOX and ASKUFH groups, the coronary reperfusion rate (defined as cessation of chest pain during the first 180’ of thrombolysis, rapid reduction of ST-segment elevation by more than 50% of the initial value within the first 180’ and rapid increase in plasma CK and CK-MB with a peak in the first 12 h) was comparable. Also 30-day mortality was comparable in both groups and neither significant difference in the incidence of major or minor hemorrhage was observed.

## CUMULATIVE ANALYSIS OF CLINICAL TRIALS

An overview of the clinical studies of LMWH as an adjunct to thrombolysis for STEMI has been recently published [17]. Although highly different in design, thrombolytic agent and LMWH used, these trials generally show a favourable effect of dalteparin, reviparin and enoxaparin on the clinical efficacy end-points, not only in comparison to placebo [5, 6, 8, 9, 14], but also to UFH [15, 11-13, 15, 16] (Table **1**). Whereas mortality is not generally influenced by the treatment with LMWH (with the only exception of the CREATE study [8], where death rate was significantly reduced at both 7 and 30 days with reviparin as compared to placebo), the occurrence of reinfarction and recurrent ischemia/angina is in general reduced, as compared to both placebo [6, 8, 9, 14] and UFH [11, 12, 15] (Table **1**). The different definitions of recurrent ischemia/angina and reinfarction, as well as the different thrombolytic (with either fibrin-specific or non fibrin-specific agents) and both LMWH and UFH regimens, may well explain the lack of a significant positive effect which was nonetheless observed in some trials [5-7, 13, 14, 16]. Also, the known differences in the pharmacokinetic properties and anticoagulation profile might, at least in part, explain the different results obtained with different LMWH. For example, enoxaparin has about twice longer plasma half-life and twice higher anti factor Xa activity than dalteparin [18]. In addition, enoxaparin, but not dalteparin, has been shown to reduce the levels of von Willebrand factor, which in turn, are associated to a poorer prognosis at 30 days in patients with non-ST elevation acute coronary syndromes [19]. Relative to the angiographic efficacy end-points, no significant differences were observed with LMWH compared to UFH on the acute (60-90’ to 24 h) patency rate of the IRA [6, 10, 12] (Table **1**). On the other hand, late patency (4 to 10 days) and/or reocclusion rates of the IRA, were in general, favourably influenced by LMWH, although statistical significance was seldom reached [10, 14, 15] (Table **1**). Finally, the safety profile of LMWH is characterized in general, by an increased occurrence of both minor and major bleedings compared to placebo [5, 8, 14] and substantially unchanged compared to UFH [7, 10-13], with only exception of the ASSENT-3 Plus study [15] where, in a pre-hospital setting, thrombolytic treatment plus enoxaparin was associated, albeit in patients older than 75 years only, with a twice higher occurrence of intracranial hemorrhage (Table **1**).

The randomized clinical trials on LMWH as compared to either placebo or UFH as adjuncts to thrombolysis in STEMI have also been an object of a recent meta-analysis [20]. In the 4 trials where LMWH were compared to placebo, about 17,000 patients were evaluated, only non fibrin-specific thrombolytics were used, and either enoxaparin, dalteparin or reviparin were evaluated [5, 6, 8, 14]. Treatment with LMWH reduced the occurrence of reinfarction and death by about one quarter and 10% during both hospitalization/at 7 days and at 1 month. Fig. (**1**) [20]. A nonsignificant excess of (hemorrhagic) stroke was observed with LMWH both during hospitalization/at 7 days (OR 1.19; 95% CI 0.84-1.70) and at 30 days (OR 1.19; 95% CI 0.86-1.65), whereas the increase of in-hospital/at 7 days increase of both major (OR 2.70; 95% CI 1.83.3.99) and minor (OR 3.24; 95% CI 2.12-4.91) bleeds was significant [20]. LMWH were compared to UFH in 6 trials, where about 7,000 patients were enrolled, both fibrin- and non fibrin-specific thrombolytics were used, and enoxaparin and dalteparin evaluated [7, 10-13, 15]. During both hospitalization/at 7 days and 30 days, the reinfarction rate was significantly reduced by LMWH, whereas the decrease in death was not significant. Fig. (**2**) [20]. There was also a nonsignificant increase of stroke (OR 1.38; 95% CI 0.95-2.01) and intracranial hemorrhage (OR 1.18; 95% CI 0.74-1.87) during hospitalization/at 7 days, which persisted at 30 days for stroke (OR 1.19; 95% CI 0.79-1.91) [19]. In-hospital/at 7 days bleeding rate was increased with LMWH, although statistical significance was reached only for minor (OR 1.26; 95% CI 1.12-1.43) and not for major (OR 1.30; 95% CI 0.98-1.72) events [20].

Therefore, according to the cumulative analysis of currently available trials, LMWH as an adjunct to thrombolysis in STEMI appear more effective than placebo and at least as effective as (if not superior to) UFH. The higher efficacy of LMWH as compared to UFH observed in some studies on the occurrence of reinfarction, may in fact not be real, since it may derive from different durations (longer with LMWH) of heparin treatments [7, 11, 12, 15]. Indeed, no differences on the occurrence of reinfarction and recurrent angina were observed when LMWH and UFH were given for the same time period [13]. Also, it should be taken into account that UFH may have been underdosed, as in ASSENT-3 [11] and ASSENT-3 Plus [15] trials, where the target aPTTs resulted below-range in about half of the patients, therefore possibly resulting in lower antithrombotic efficacy of UFH.

To summarize, clinical evidence from the trials reviewed above supports the use of LMWH (essentially enoxaparin) as a valid alternative (if not the treatment to be preferred) to UFH as an adjunct to thrombolysis young (< 75 years old) STEMI patients without renal dysfunction. Whereas sufficient evidence does not exist to make this recommendation for dalteparin, further and stronger data in favour of enoxaparin have been recently obtained for the ExTRACT-TIMI 25 trial [21]

## THE ExTRACT-TIMI 25 TRIAL

In this multi-center, double-blind, randomized trial, over 20.000 patients receiving either a fibrin- or non fibrin-specific thrombolytic and aspirin, were randomized to intravenous bolus of UFH 60 IU/kg followed by infusion of 12 IU/kg/h for 48 hours, or enoxaparin 30 mg as an intra-venous bolus followed by 1 mg/kg twice daily subcutaneously for up to 8 days [21]. In patients at least 75 years of age the intravenous bolus was eliminated and the subcutaneous dose reduced to 0.75 mg/kg every 12 hours, whereas in patients with an estimated creatinine clearance of less than 30 ml/min the dose was modified to 1 mg/kg every 24 hours [21]. The primary end-point was the composite of death or reinfarction at 30 days, whereas secondary end points were the composite of death and reinfarction/recurrent ischemia and the composite of death, recurrent reinfarction and disabling stroke at 30 days. Enoxaparin was significantly more effective than UFH on both in-hospital (7% vs 9%; p<0.001) and 30-day (10% vs 12%; p<0.001) primary end point. Fig. (**3**) and Fig. (**4**). In both cases, this result was mainly driven by the significant decrease in reinfarction, since mortality was not substantially affected. Fig. (**3**) and Fig. (**4**). At 30 days, major bleedings were significantly more frequent with enoxaparin (in spite of the dose-adjustments according to age and renal function) (2.1% vs 1.4%; p<0.001), although the occurrence of intracranial hemorrhage was comparable (0.8% vs 0.7%) Fig. (**4**). However, the net clinical benefit at 30 days, defined as the combined occurrence of death, reinfarction and either nonfatal disabling stroke, major bleeding or intracranial hemorrhage, was significantly higher with enoxaparin, which was associated with a significant 14 to 18% relative risk reduction of these events compared to UFH. Because of its design and size, the ExTRACT-TIMI 25 study [21] should be considered conclusive about the superior efficacy of enoxaparin in comparison to UFH for the treatment of patients receiving thrombolysis for STEMI. Again however, it cannot be determined whether this result is to be ascribed to a true superior antithrombotic effect of enoxaparin or instead to the longer duration of treatment (7 days vs 48 hours). Also, the higher occurrence of major bleedings may also well ascribed to the longer duration of treatment, rather than to a superior dangerousness of enoxaparin. Since however, the most dreadful and disabling hemorrhagic complication, represented by intracranial bleeding, did not significantly differ in the two groups, the safety profile of enoxaparin should be considered satisfactory.

## CONCLUSIONS

The administration of LMWH as an adjunct to thrombolysis with either fibrin- and non fibrin-specific agents in STEMI patients appears at least as effective as safe as UFH. Because of the easiness of subcutaneous administration and the lack of need for aPTT monitoring they should be strongly considered in this clinical setting, since prolonged antithrombotic treatment is possible, without hampering early mobilization and rehabilitation of patients. Because enoxaparin has been the most extensively studied LMWH and has most consistently shown a superiority to both placebo and UFH on both in-hospital and 30-day occurrence of reinfarction/recurrent ischemia, and angiographic end-points, such as patency and reocclusion rates of the IRA, it should be considered the compound of choice. Provided that patients are aged less than 75 years and significant renal dysfunction is absent, enoxaparin should be administered as a 30-mg intravenous bolus immediately prior to thrombolytic administration followed by 1 mg/kg subcutaneously twice daily for about 1 week. Before definitively replacing UFH however, some important, and yet unresolved issues, such as the use in elderly patients, and in conjunction with glycoprotein IIb/IIIa inhibitors and percutaneous coronary interventions, need to be addressed.

## Figures and Tables

**Fig. (1):**
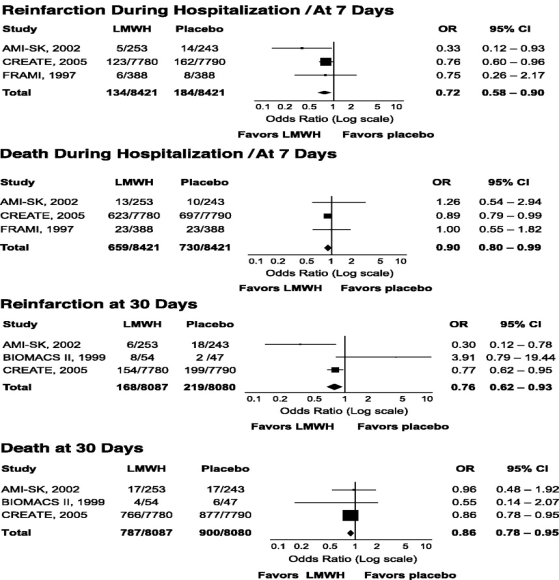
Efficacy of low-molecular-weight heparins (LMWH) as compared to placebo in 4 randomized clinical trials (from reference [19]).

**Fig. (2):**
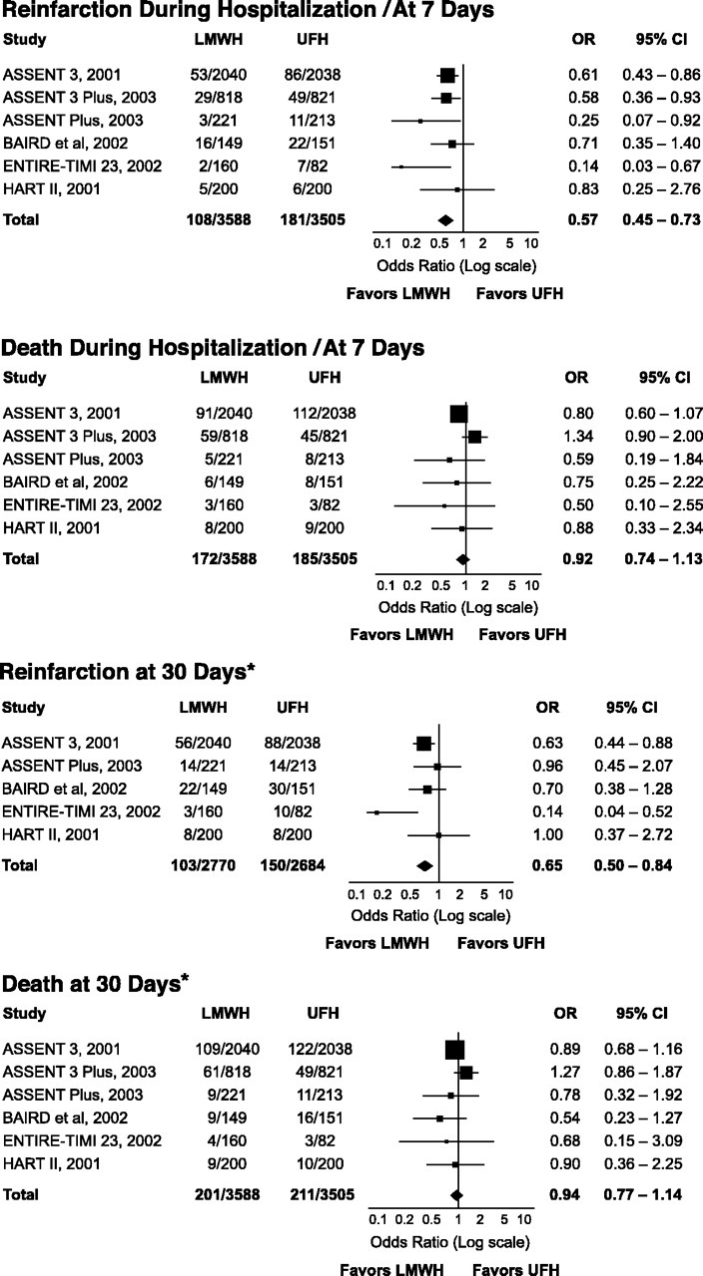
Efficacy of low-molecular-weight heparins (LMWH) as compared to unfractionated heparin (UFH) in 6 randomized clinical trials (from reference [19]). * Outcome for Baird *et al.* [12] is at 90 days.

**Fig. (3):**
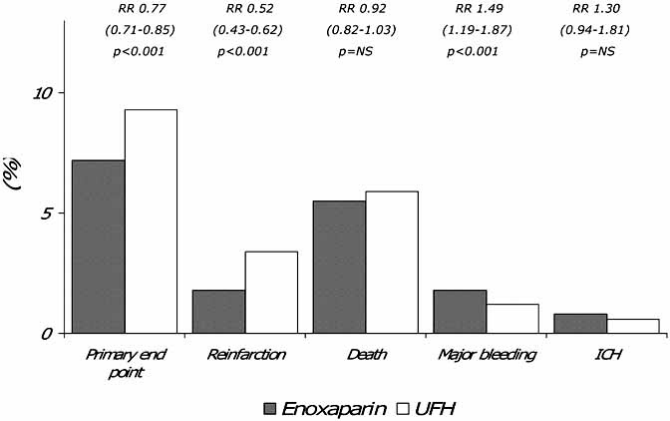
Efficacy and safety outcomes at 8 days in the ExTRACT-TIMI 25 study [20]. UFH = unfractionated heparin; ICH = intracranial hemorrhage; RR = relative risk; NS = nonsignificant.

**Fig. (4):**
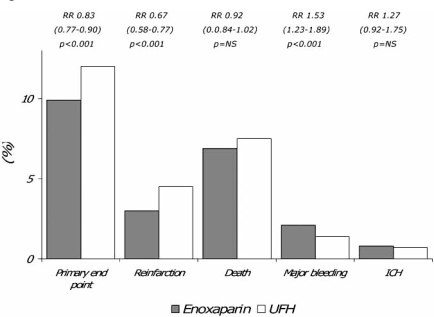
Efficacy and safety outcomes at 30 days in the ExTRACT-TIMI 25 study [20]. UFH = unfractionated heparin; ICH = intracranial hemorrhage; RR = relative risk; NS = nonsignificant.

**Table 1:** Clinical Trials with Dalteparin, Enoxaparin and Reviparin

Study	N° Pts		Treatment		Treatment Duration	End points	Results (%) Active* vs* Control
		Active		Control			
Dalteparin							
FRAMI [4]	776		SK + ASA +		9 days	1°: LV thrombus, arterial embolism at 9 days	14–22*
		D 150 IU/kg sc x 2		placebo		2°: death/myocardial reinfarction at 3 months	
BIOMACS II [5]	101		SK + ASA +		20-28 h	1°: TIMI grade 3 flow in IRA at 24 hours	2–2/6 - 6
		D 100 IU/Kg sc +		placebo +		2°: ischemic ECG episodes at 1/21 days	68-51
		only D 120 IU/kg sc 12 h later					
ASSENT [6] PLUS	439		rt-PA + ASA +		4-7 days D/48 h UFH	1°: in-H TIMI grade 3 flow in IRA	69-63
		D 120 IU/kg ¼ dose iv +		UFH 4000/5000 IU iv +		2°: death at 7/30 days	2.3-3.8/4.1–5.2
		D 120 IU/kg ¼ dose sc +		UFH 800/1000 IU/h iv		2°: myocardial reinfarction at 7/30 days	1.4–5.4*/6.5-7
		D 120 IU/kg sc x 2				2°: major bleeding at 30 days	3.7–4.6
Reviparin							
CREATE [7]	15570		Thrombolytic + ASA +		7 days	1°: death, reinfarction, stroke at 7 days	9.6–11*
		<50Kg R 3436IU sc x 2		placebo		1°: previous + recurrent ischemia at 7 days	11.1–12.6*
		50-75Kg R 5153IU sc x 2		placebo		2°: death, reinfarction, stroke at 30 days	11.8–13.6*
		>75Kg R 6871IU sc x 2		placebo		2°: previous + recurrent ischemia at 30 days	13.8–15.6*
						2°: major bleeding at 7 days	0.9–0.4*
Enoxaparin							
Glick *et al.* [8]	103		SK + ASA + UFH iv for 5 days +		25 days	1°: death, reinfarction, angina at 6 months	14–43*
		E 40mg/die sc		placebo			
HART-II [9]	400		rt-PA + ASA +		3 days	1°: TIMI 2-3 grade flow in IRA at 90’	80-75
		E 30mg iv +		UFH 5.000 IU iv +		2°: in-H major bleeding	5.6-5
		E 1mg/kg sc x 2		15 IU/kg/h iv			
ASSENT-3 [10]	6095	full-dose TNK + ASA +		full-dose TNK + ASA +	7 days E/48 h UFH	1°: death at 30 days, in-H reinfarction and angina	11–15*
		E 30mg iv + 1mg/Kg sc x 2		UFH 60 IU/kg iv +12 IU/kg/h iv		1°: previous + in-H major bleeding	14–17*
				half-dose TNK + ASA +		2°: in-H reinfarction/refractory ischemia	3–4*/5–7*
				UFH 40 IU/kg +7 IU/kg iv + abciximab 12 h		2°: in-H major bleeding	3–2
ENTIRE [11]	483		as in ASSENT–3 trial +		≤ 8 days E/≤ 36 h UFH	1°: TIMI grade 3 flow at 60’	50-51
TIMI 23		half-dose TNK + E 1 mg/kg sc x 2 + abciximab 12 h				1°: major bleeding at 30 days	2–2
						2°: death, reinfarction at 30 days	4–16*
Baird *et al*. [12]	300		Thrombolytic +		4 day	1°: death, reinfarction, angina at 90 days	26–36*
		E 40mg iv +		UFH 5.000 IU iv +		2°: major bleeding at 4 days	3-4
		E 40mg sc x 3		UFH 30.000 IU/24 h			
AMI-SK [13]	496	+	SK + ASA		3-8 days	1°: TIMI 3 grade flow in IRA at 5-10 days	70–58*
		E 30 mg iv +		placebo		2°: death, reinfarction, angina at 30 days	13–21*
		E 1 mg/kg sc x 2				2°: major bleeding at 30 days	5-3
ASSENT-3 [14]	1639		TNK + ASA +		≤ 7 days E/48 h UFH	1°: death at 30 days, in-H reinfarction and angina	14-17
PLUS		E 30mg iv +		UFH 60 IU/kg +12 IU/kg/h iv		1°: previous + in-H major bleeding	18-20
		E 1 mg/kg sc x 2				2°: stroke + intracranial hemorrhage	2-1
ASENOX [15]	429		ASK + ASA +		≤ 7 days E/≤ 72 h UFH	1°: Reperfusion rate (noninvasive criteria)	77.6–73.5
		E 40 mg iv + 1 mg/kg sc x 2		UFH 1000 IU/h		2°: mortality at 30 days	6.1–6.8

1°: primary end-point; 2°: secondary end-point; D: dalteparin; E: enoxaparin; R: reviparin; rt-PA: alteplase; SK: streptokinase; ASK: accelerate regimen of streptokinase in 20 min; TNK: tenecteplase; ASA: aspirin; LMWH: low molecular weight heparin; UFH: unfractionated heparin; IRA: infarct-related artery; LV: left ventricle; sc: subcutaneously; iv: intravenously; H: hospital.

* p < 0.05.

## References

[1] Cheng JWM (2001). Recognition, pathophysiology, and management of acute myocardial infarction. Am J Health Syst Pharm.

[2] Ewald GA, Eisenberg PR (1995). Plasmin-mediated activation of contact system in response to pharmacological thrombolysis. Circulation.

[3] Antman EM, Anbe DT, Armstrong PW (2004). ACC/AHA guidelines for the management of patients with ST-elevation myocardial infarction: executive summary and recommendation: a report of the American College of Cardiology/American Heart Association Task Force on practice guidelines. Circulation.

[4] Van de, Werf F, Ardissino D, Betriu A (2003). Management of acute myocardial infarction in patients presenting with ST-segment elevation. Eur Heart J.

[5] Kontny F, Dale J, Abildgaard U, Pedersen TR (1997). Randomized trial of low molecular weight heparin (Dalteparin) in prevention of left ventricular thrombus formation and arterial embolism after acute anterior myocardial infarction: the Fragmin in Acute Myocardial Infarction (FRAMI) study. J Am Coll Cardiol.

[6] Frostfeldt G, Ahlberg G, Gustafsson G (1999). Low molecular weight heparin (Dalteparin) as adjuvant treatment to thrombolysis in acute myocardial infarction – a pilot study: Biochemical Markers in Acute Coronary Syndromes (BIOMACS II). J Am Coll Cardiol.

[7] Wallentin L, Bergstrand L, Dellborg M (2003). Low molecular weight heparin (dalteparin) compared to unfractionated heparin as an adjunct to rt-PA (alteplase) for improvement of coronary artery patency in acute myocardial infarction – the ASSENT Plus study. Eur Heart J.

[8] The CREATE Trial Group Investigators (2005). Effects of reviparin, a low-molecular-weight heparin, on mortality, reinfarction, and strokes in patients with acute myocardial infarction presenting with ST-segment elevation. JAMA.

[9] Glick A, Kornowski R, Michowich Y (1996). Reduction of reinfarction and angina with use of low-molecular-weight heparin therapy after sterptokinase (and heparin) in acute myocardial infarction. Am J Cardiol.

[10] Ross AM, Molhoek P, Lundergan C (2001). Randomized comparison of enoxaparin, a low-molecular-weight heparin, with unfractioned heparin adjunctive to recombinant tissue plasminogen activator thrombolysis and aspirin. Second trial of Heparin and Aspirin Reperfusion Therapy (HART-II). Circulation.

[11] (2001). The Assessment of the Safety and Efficacy of a New Thrombolytic regimen (ASSENT)-3 investigators. Efficacy and safety of tenecteplase in combination with enoxaparin, abciximab, or unfractioned heparin: the ASSENT-3 randomised trial in acute myocardial infarction. Lancet.

[12] Antman EM, Louwerenburg HW, Baars HF (2002). Enoxaparin as adjunctive antithrombin therapy for ST-elevation myocardial infarction. Results of the ENTIRE-Thrombolysis In Myocardial Infarction (TIMI) 23 Trial. Circulation.

[13] Baird SH, Menown IBA, McBride SJ, Trouton TG, Wilson C (2002). Randomized comparison of enoxaparin with unfractioned heparin following fibrinolytic therapy for acute myocardial infarction. Eur Heart J.

[14] Simoons ML, Krzeminska-Pakula M,  Alonso A (2002). Improved reperfusion and clinical outcome with enoxaparin as an adjunct to streptokinase thrombolysis in acute myocardial infarction. The AMI-SK Study. Eur Heart J.

[15] Wallentin L, Goldstein P, Armstrong PW (2003). Efficacy and safety of tenecteplase in combination with the low-molecular-weight heparin enoxaparin or unfractionated heparin in the prehospital setting. The Assessment of the Safety and Efficacy of a New Thrombolytic Regimen (ASSENT)-3 PLUS randomized trial in acute myocardial infarction. Circulation.

[16] Tatu-Chitoiu G, Teodorescu C, Capraru P (2004). Accelerated streptokinase and enoxaparin in ST-segment elevation acute myocardial infarction (the ASENOX study). Pol Heart J.

[17] Rubboli A, Ottani F, Capecchi A, Brancaleoni R, Galvani M, Swahn E (2006). Low-molecular-weight heparins in conjunction with thrombolysis for ST-elevation acute myocardial infarction. A critical review of the literature. Cardiology.

[18] Collignon F, Frydman A, Caplain H (1995). Comparison of the pharmacokinetic profiles of three low molecular weight mass heparins–dalteparin, enoxaparin and nadroparin–administered subcutaneously in healthy volunteers (doses for prevention of thromboembolism). Thromb Haemost.

[19] Montalescot G, Collet JP, Lison L (2000). Effects of various anticoagulant treatments on von Willebrand factor release in unstable angina. J Am Coll Cardiol.

[20] Eikelboom JW, Quinlan DJ, Mehta SR, Turpie AG, Menown IB, Yusuf S (2005). Unfractionated and low-molecular-weight heparin as adjuncts to thrombolysis in aspirin-treated patients with ST-elevation acute myocardial infarction. A meta-analysis of the randomized trials. Circulation.

[21] Antman EM, Morrow DA, McCabe CH (2006). Enoxaparin versus unfractionated heparin with thrombolysis for ST-elevation myocardial infarction. N Engl J Med.

